# Inhibitory Effect of S0859 on the Antioxidant Master Switch Nuclear Factor Erythroid 2-Related Factor 2 in Lung Cancer Cells

**DOI:** 10.3390/antiox14101191

**Published:** 2025-09-28

**Authors:** Eunsun Lee, Jeong Hee Hong

**Affiliations:** Department of Physiology, College of Medicine, Lee Gil Ya Cancer and Diabetes Institute, Gachon University, 155 Getbeolro, Yeonsu-gu, Incheon 21999, Republic of Korea; eunsunpp@gachon.ac.kr

**Keywords:** NBCn1, NRF2, oxidative stress, S0859, NF-κB

## Abstract

Cancer cells possess endogenous antioxidant systems such as nuclear factor erythroid 2-related factor 2 (NRF2). The electroneutral sodium bicarbonate cotransporter NBCn1, known as a migratory module, is closely associated with cancer metastasis; however, its regulatory signaling in cancer remains unclear. In particular, the regulation of NBCn1 in response to oxidative stress and its relationship with NRF2 need to be elucidated. In the present study, we found that hydrogen peroxide–induced oxidative stress dysregulated NBCn1 via inhibition of NF-κB, thereby suppressing cellular migration in non-small cell lung cancer A549 cells. Phosphorylation of NF-κB was required for maintaining NBCn1 function in A549 cells. Oxidative stress also induced NRF2 nuclear translocation, reduced NBC activity, and activated oxidative stress–responsive gene expression. Treatment with the NBC inhibitor S0859 impaired ERK activation, NRF2 nuclear translocation, and oxidative stress defense gene expression in A549 cells. Furthermore, oxidative stimulation in the presence of S0859 disrupted the NRF2-mediated oxidative stress defense system and cellular migration in A549 lung cancer cells. Collectively, these findings suggest that S0859, as a potential NRF2 inhibitor, may exert anti-cancer properties.

## 1. Introduction

Sodium-bicarbonate cotransporter n1 (NBCn1) is considered a migratory module involved in cancer metastasis [1,2]. NBCn1 is commonly known as a plasma membrane-associated pH regulator that transfers bicarbonate with sodium ions and has been mainly studied in the modulation of cellular pH homeostasis through bicarbonate absorption to maintain bicarbonate-associated systemic regulation, such as compensation of respiratory acidosis in renal proximal tubules [3,4]. Moreover, we previously reported that NBCn1 is expressed in lung cancer cells and is involved in lung cancer cell migration [5].

The nuclear factor erythroid 2-related factor 2 (NRF2) is a master anti-oxidative transcription factor to respond to oxidative stimuli through the regulation of anti-oxidative genes and enzymes, including heme oxygenase-1 (HO-1) [6]. Generally, cancer cells possess overexpressed antioxidant systems to tolerate the oxidative stress-enriched circumstances better than normal cells. Thus, lung cancer cells possess several oxidative stress defense systems, including NRF2 [7,8]. Its response to oxidative stress reveals diversity, dependent on the lung cancer cell types, and its dual role in cancer progression or inhibition is dependent on cancer type or stage [8,9]. Although debate about the dual role of NRF2 has occurred, stimulation of oxidative stress induces the activation of NRF2 to initiate the anti-oxidative process in lung cancer [10,11]. The activation of NRF2 is known to be involved in cancer development and drug resistance [6,7,8,12]. For instance, oxidative stress induces NRF2 activation and subsequently mediates the transcription of various NRF2 target gene sets such as antioxidant, lipid metabolism, heme/iron metabolism, autophagy, and apoptosis [13,14].

Previously, we addressed that acute stimulation of hydrogen peroxide induces the activation of both NBCn1 and the electrogenic type of NBC, NBCe1-B, in human embryonic kidney 293T (HEK293T) cells [15]. NBCn1 is closely related to cancer development, such as metastasis and pH regulation [5]. Oxidative stress is known to profoundly influence ion transport mechanisms; however, the role of NBCn1 under such conditions has not been clearly defined in lung cancer cells. Given that the antioxidant regulator NRF2 is intricately linked with NF-κB [16], a pivotal transcriptional regulator of stress-responsive genes, we postulate that NBCn1 functions as a key ion transporter whose expression and/or activity is modulated by NF-κB signaling during oxidative stress in lung cancer cells. Furthermore, we hypothesize that the crosstalk between NF-κB and NRF2 coordinately regulates NBCn1, thereby integrating antioxidant signaling pathways and suggesting that NBCn1 inhibition might represent a novel strategy to treat lung cancer cells.

In this study, we determined the modulation of NBCn1 through NBC inhibitor S0859 in the presence of hydrogen peroxide-mediated oxidative stress and the regulatory role of NRF2 and the relationship between NBCn1 and NRF2 in lung cancer cells to verify the cellular mechanism of NRF2-associated network and to support expanding the developmental anti-cancer strategy against NSCLC. Moreover, we determined the modulatory effect of NBCn1 on NRF2 signaling as a therapeutic strategy.

## 2. Materials and Methods

### 2.1. Cell Culture

The A549 and H1299 human lung adenocarcinoma and the HEK293T cell lines were obtained from the American Type Culture Collection (ATCC, Rockville, MD, USA). Cells were cultured in Dulbecco’s Modified Eagle’s Medium (DMEM; #11995-065, Invitrogen, Waltham, MA, USA) supplemented with 10% fetal bovine serum (FBS; #16000-044, Invitrogen, Waltham, MA, USA), and 100 U/mL penicillin–streptomycin (P/S; #15140-122, Invitrogen, Waltham, MA, USA). All cells were incubated at 37 °C in a humidified incubator with 5% CO_2_ and 95% air. For fluorescence imaging, coverslips were placed in the culture dishes prior to seeding. To passage the cells, monolayers were washed with Dulbecco’s phosphate-buffered saline (DPBS; #LB001-02, Welgene, Gyeongsan, Republic of Korea) and detached using trypsin-EDTA (0.05% trypsin, 0.53 mM EDTA; #25200-072, Invitrogen, Waltham, MA, USA).

### 2.2. Genetic Manipulation and Transfection

To modulate gene expression, A549 cells were transfected with NRF2-targeting small interfering RNA (siNRF2; #sc-37030, Santa Cruz Biotechnology, Dallas, TX, USA) to induce gene knockdown, while HEK293T cells were transfected with pcDNA3.1-NBCn1 plasmid DNA (plasmid #80989, Addgene, Watertown, MA, USA) to overexpress the sodium bicarbonate cotransporter NBCn1. Transfections were performed using Lipofectamine^®^ 2000 (#11668-019, Invitrogen, Carlsbad, CA, USA) according to the manufacturer’s protocol. siRNA or plasmid DNA was diluted in OPTI-MEM medium (#31985-070, Invitrogen, Carlsbad, CA, USA) and mixed with Lipofectamine reagent, followed by a 10 min incubation at room temperature. The transfection complexes were then added to the cells and incubated for 6 h, after which the medium was replaced with fresh complete DMEM. Cells were harvested at either 24 or 48 h post-transfection, depending on the experimental requirements.

### 2.3. Transwell Membrane Migration Assay

Cellular migration was assessed using transwell inserts with polycarbonate membranes (6.5 mm insert, 8.0-μm pore size; #3422, Corning, NY, USA). The upper chambers were seeded with 5 × 10^4^ of A549 or HEK293T (NBCn1) cells suspended in 200 μL of low-serum (1% FBS) medium. A549 cells were transfected with or without siNRF2, while HEK293T cells were used without additional genetic modification. The lower chambers contained 500 μL of DMEM supplemented with hydrogen peroxide (H_2_O_2_; 10μM; #UN2014, Duksan, Daejeon, Republic of Korea) or a combination of H_2_O_2_ and S0859 (40 μM; #SML0365, Sigma-Aldrich, St. Louis, MO, USA). After 1 h incubation, cells on the upper surface of the membrane were removed, and the membranes were fixed with cold methanol (#34860, Sigma-Aldrich, St. Louis, MO, USA) at −20 °C for 1 min. After washing with phosphate-buffered saline (PBS; #10010-023, Gibco, Grand Island, NY, USA), membranes were counterstained with DAPI (#62248, Thermo Fisher Scientific, Waltham, MA, USA) for 30 min in the dark. DAPI-stained nuclei were visualized at 405 nm using an LSM 700 confocal microscope (LSM700, Zeiss, Oberkochen, Germany), and migrated cells were quantified based on the intensity of nuclei.

### 2.4. Quantitative Real-Time PCR (qRT-PCR)

Total RNA was extracted from A549 cells using RiboEx reagent (GeneAll, Seoul, Republic of Korea), followed by chloroform-based phase separation and centrifugation at 12,000× *g* for 15 min at 4 °C. The aqueous phase was purified using the Hybrid-R RT-PCR kit (GeneAll, Seoul, Republic of Korea) according to the manufacturer’s instructions, and RNA concentration was determined using an ND-1000 spectrophotometer (Thermo Fisher Scientific, Waltham, MA, USA). Complementary DNA was synthesized from 1 μg of RNA using AccuPower^®^ RocketScript™ Cycle RT PreMix (Bioneer, Daejeon, Republic of Korea). Quantitative real-time PCR (qRT-PCR) was performed using PowerUp™ SYBR™ Green Master Mix (#A25741, Applied Biosystems, Waltham, MA, USA) on a QuantStudio™ 3 Real-Time PCR System (#A28567, Applied Biosystems, Waltham, MA, USA). Gene-specific primers were custom-synthesized by BIONICS Oligo (Bionics Co., Seoul, Republic of Korea), and the sequences were as follows: GAPDH forward 5′-GAC CTG ACC TGC CGT CTA GAA A-3′ and reverse 5′-CCT GCT TCA CCA CCT TCT TGA-3′; *SLC4A7* forward 5′-AAT TCC TAC GGG TGC TGA GG-3′ and reverse 5′-GTA AGG AGG ACA GCA GGA GC-3′; *SQSTM1* (encoding p62 protein) forward 5′-GGC AGT AGG CAC CAC CAT GTA-3′ and reverse 5′-GAT TCA ACC GCC ATG TGC TT-3′; *HMOX1* (encoding HO-1 protein) forward 5′-GCA GCT GTC TCA AAC CTC CAA-3′ and reverse 5′-AGT GGT CAT GGC CGT GTC A-3′. GAPDH was used as the internal control. The cycling conditions were as follows: uracil-DNA glycosylase (UDG) activation at 50 °C for 2 min, dual-lock DNA polymerase activation at 95 °C for 2 min, followed by 40 cycles of denaturation at 95 °C for 15 s, annealing at 55 °C for 15 s, and extension at 72 °C for 1 min.

### 2.5. Western Blotting

Cells (2 × 10^5^ per well) were seeded into six-well plates and incubated at 37 °C. After treatment, cells were lysed using immunoprecipitation assay buffer (#R0278, Sigma-Aldrich, St. Louis, MO, USA) supplemented with protease inhibitor (P8340, Sigma-Aldrich, St. Louis, MO, USA) and phosphatase inhibitor cocktails (#P5726, Sigma-Aldrich, St. Louis, MO, USA). Protein concentrations were determined using the Bradford assay (#5000205, Bio-Rad, Hercules, CA, USA). Equal amounts of protein (30 μg) were separated by SDS–PAGE, transferred to PVDF membranes (#IPVH00010, Millipore, Burlington, MA, USA), and incubated with 5% skim milk powder-included blocking buffer (#70166, Sigma-Aldrich, St. Louis, MO, USA) in TBS-T. Membranes were incubated overnight at 4 °C with the following primary antibodies: NBCn1 (#ab82335, Abcam, Cambridge, UK), NRF2 (#14409, Cell Signaling Technology, Danvers, MA, USA), nuclear factor-kappa B (NF-κB; #8242, Cell Signaling Technology, Danvers, MA, USA), phospho-NF-κB (p-NF-κB; #3033, Cell Signaling Technology, Danvers, MA, USA), extracellular signal-regulated kinase (ERK; #4695, Cell Signaling Technology, Danvers, MA, USA), and phospho-ERK (p-ERK; #4370, Cell Signaling Technology, Danvers, MA, USA). After washing, membranes were incubated with HRP-conjugated secondary antibodies (anti-rabbit IgG, #7074, Cell Signaling Technology, Danvers, MA, USA) and detected using enhanced chemiluminescence reagents (#32106, Thermo Fisher Scientific, Waltham, MA, USA). Images were captured with an Amersham ImageQuant 800 imaging system (Cytiva, Marlborough, MA, USA). Pre-stained protein molecular weight markers (PageRuler™ Prestained Protein Ladder, #26616, Thermo Fisher Scientific, Waltham, MA, USA; and Precision Plus Protein™ Dual Color Standards, #161-0374, Bio-Rad, Hercules, CA, USA) were used to estimate protein sizes.

### 2.6. Immunofluorescence Staining

A549 cells were cultured on coverslips in six-well plates and treated with or without H_2_O_2_ (10 μM), siNRF2, or S0859 (40 μM). Cells were fixed with 4% paraformaldehyde (#15710, Electron Microscopy Sciences, Hatfield, PA, USA) for 15 min, permeabilized with 0.1% Triton X-100 (#T8787, Sigma-Aldrich, St. Louis, MO, USA) for 10 min, and blocked with 0.5% bovine serum albumin (BSA; #A9647, Sigma-Aldrich, St. Louis, MO, USA) for 1 h. Primary antibodies used were against NBCn1, NRF2, p-NF-Κb, or LAMIN A/C (#ab185014, Abcam, Cambridge, UK). After washing, fluorescence-labeled secondary antibodies were applied for 1 h at room temperature in the dark: rhodamine-tagged goat IgG (Jackson ImmunoResearch, West Grove, PA, USA; anti-mouse: #115-025-072, anti-rabbit: #111-025-144) and fluorescein isothiocyanate (FITC)-conjugated goat IgG (Jackson ImmunoResearch, West Grove, PA, USA; anti-mouse: #115-095-071, anti-rabbit: #111-095-003). Coverslips were mounted with Fluoromount G-containing DAPI solution (#17984-24, Electron Microscopy Sciences, Hatfield, PA, USA) and imaged using an LSM 700 confocal microscope (Zeiss, Oberkochen, Germany). Representative images were obtained.

### 2.7. Intracellular pH Measurement for NBC Activity

Cells were cultured on coverslips and incubated with the pH-sensitive fluorescent dye 2′,7′-bis-(carboxyethyl)-5-(and-6)-carboxyfluorescein acetoxymethyl ester (BCECF-AM; 20 μM, #B1170, Invitrogen, Carlsbad, CA, USA) along with 0.1% Pluronic F-127 (#P3000MP, Invitrogen, Carlsbad, CA, USA) for 15 min at room temperature. Following dye loading, cells were perfused with CO_2_-saturated, bicarbonate-buffered physiological salt solution (composition in mM: 120 NaCl, 10 D-glucose, 5 KCl, 1 MgCl_2_, 1 CaCl_2_, 2.5 HEPES, and 25 NaHCO_3_; pH 7.8) for at least 5 min. Sodium/hydrogen exchanger (NHE) activity was inhibited by supplementing all solutions with 10 μM 5-(N-ethyl-N-isopropyl) amiloride (EIPA; #A3085, Sigma-Aldrich, St. Louis, MO, USA). Intracellular pH (pH_i_) was measured by detecting BCECF fluorescence using dual excitation wavelengths (440 nm and 495 nm) and an emission wavelength at 530 nm. NBC activity was evaluated by acid-loading the cells using a sodium-free, bicarbonate-buffered solution in which NaCl was replaced with equimolar N-methyl-D-glucamine (NMDG, 99%; #126840025, Acros Organics, Geel, Belgium). The composition of the solution was (in mM): 120 NMDG-Cl, 5 KCl, 1 MgCl_2_, 1 CaCl_2_, 2.5 HEPES, and 25 NaHCO_3_ (pH 7.8). After acid-loading, pH_i_ recovery rate was monitored to assess NBC activity. Fluorescence images were acquired using a Retiga 6000 CCD camera (Q-Imaging, Surrey, BC, Canada) mounted on an inverted microscope (IX73, Olympus, Tokyo, Japan) and analyzed using the MetaFluor imaging system (Molecular Devices, San Jose, CA, USA). Background fluorescence was subtracted individually from each image. NBC activity was quantified as the slope of pH_i_ recovery rate (ΔpH/sec).

### 2.8. MTT Assay for Cell Viability

Cell viability was assessed using the 3-(4,5-dimethylthiazol-2-yl)-2,5-diphenyltetrazolium bromide (MTT) assay. A549 and H1299 cells (1 × 10^4^ per well) were seeded into 96-well plates and treated with the indicated condition depending on the experimental group. After treatment, 20 μL of MTT solution (2 mg/mL; #M2128, Sigma-Aldrich, St. Louis, MO, USA) was added per well and incubated at 37 °C for 2 h. Formazan crystals were solubilized in 100% dimethyl sulfoxide (DMSO; #D8418, Sigma-Aldrich, St. Louis, MO, USA), and absorbance was measured at 570 nm using a Varioskan LUX microplate reader (Thermo Fisher Scientific, Waltham, MA, USA).

### 2.9. Statistical Analysis

Data are presented as the mean ± standard error of the mean (SEM) from at least three independent experiments. Statistical comparisons were made using one-way ANOVA followed by Tukey’s post hoc test or other appropriate post hoc tests. A *p*-value < 0.05 was considered statistically significant. Significance is indicated as * *p* < 0.05, ** *p* < 0.01, *** *p* < 0.001 vs. control; # *p* < 0.05, ## *p* < 0.01, ### *p* < 0.001 vs. H_2_O_2_.

## 3. Results

### 3.1. H_2_O_2_ Reduces NBCn1 Expression and Impairs Cellular Migration in A549 Cells

**Figure 1:**
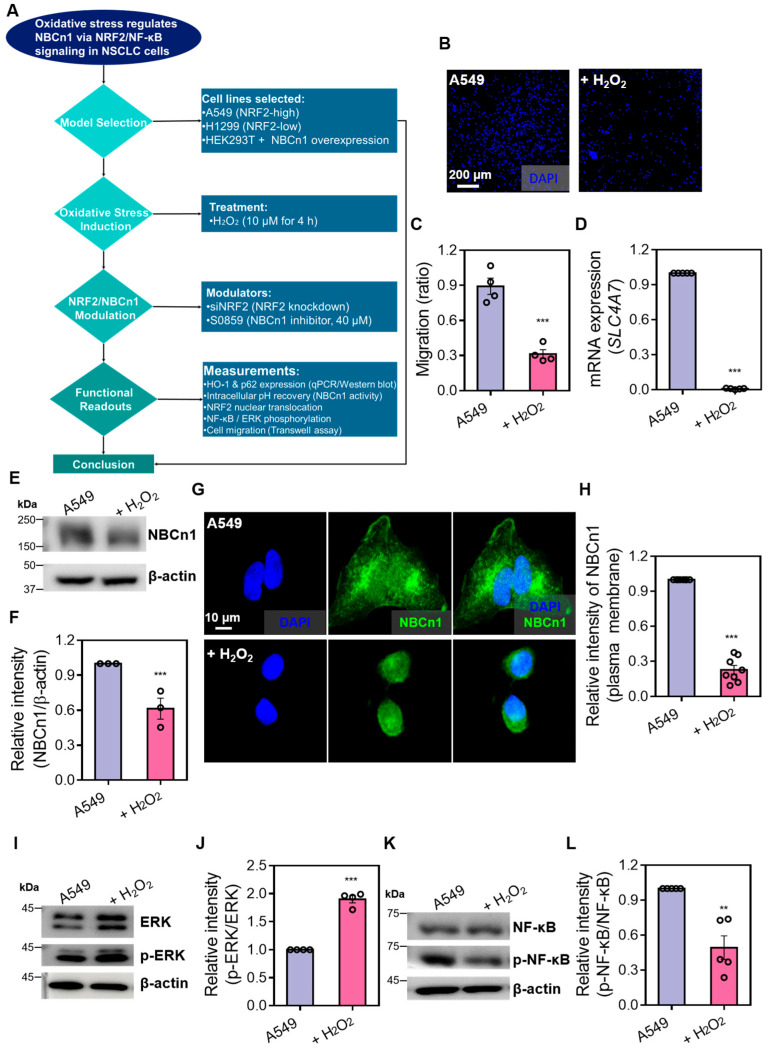
H_2_O_2_ reduces NBCn1 expression and impairs cellular migration in A549 cells. (**A**) Schematic summary of the experimental approach. (**B**) Representative images of transwell migration assays in A549 cells treated with or without H_2_O_2_ (10 μM, 1 h), stained with DAPI. Scale bar = 200 μm. (**C**) Analysis of cellular migration expressed as a relative migration ratio. Data are presented as mean ± SEM (*n* = 4, *** *p* < 0.001 vs. control). (**D**) The mRNA expression of *SLC4A7* in A549 cells treated with or without H_2_O_2_. Data are presented as mean ± SEM (*n* = 5, *** *p* < 0.001 vs. control). (**E**) Protein expression levels of NBCn1 in A549 cells with or without H_2_O_2_ treatment. β-actin was used as a loading control. (**F**) Relative intensity of NBCn1 protein normalized to β-actin. Data are presented as mean ± SEM (*n* = 3, *** *p* < 0.001 vs. control). (**G**) Immunofluorescence staining of NBCn1 (green) and nuclei (DAPI, blue) in A549 cells treated with or without H_2_O_2_. Scale bar = 10 μm. (**H**) Fluorescence intensity analysis of plasma membrane-localized NBCn1. Data are presented as mean ± SEM (*n* = 8, *** *p* < 0.001 vs. control). (**I**) Protein expression levels of total ERK and p-ERK in A549 cells treated with or without H_2_O_2_. β-actin was used as a loading control. (**J**) Relative intensity of p-ERK normalized to total ERK. Data are presented as mean ± SEM (*n* = 4, *** *p* < 0.001 vs. control). (**K**) Protein expression levels of total NF-κB and p-NF-κB in A549 cells treated with or without H_2_O_2_. β-actin was used as a loading control. (**L**) Relative intensity of p-NF-κB normalized to total NF-κB. Data are presented as mean ± SEM (*n* = 5, ** *p* < 0.01 vs. control).

To understand our hypothesis according to the crosstalk between NF-κB and NRF2 on the regulation of NBCn1, we represented the experimental approach applied in this study (Figure 1A). To evaluate the effect of oxidative stress on NBCn1 expression and cellular motility, A549 cells were treated with hydrogen peroxide (H_2_O_2_). A transwell migration assay revealed reduced cellular migration following H_2_O_2_ exposure compared to untreated control cells (Figure 1B,C). Quantitative real-time PCR analysis showed that mRNA expression of *SLC4A7*, which encodes the NBCn1 transporter, was downregulated by H_2_O_2_ treatment (Figure 1D). Consistent with the transcriptional suppression, Western blot of membrane protein fractions showed a decrease in NBCn1 protein levels upon H_2_O_2_ exposure (Figure 1E,F). Reduced NBCn1 expression was further confirmed by immunofluorescence staining, which showed diminished NBCn1 localization at the plasma membrane under oxidative stress conditions (Figure 1G,H). NF-κB, a well-characterized redox-sensitive transcription factor, and ERK, a MAPK family member modulated by ROS through upstream effectors, integrate oxidative cues to regulate cell survival and proliferation [16]. To investigate redox-sensitive signaling pathways, Western blot analysis of cellular signaling proteins was performed. Phosphorylation of ERK (p-ERK) was increased in response to H_2_O_2_ (Figure 1I,J), indicating that ERK signaling is independent of NBC activation. In contrast, p-NF-κB was reduced following H_2_O_2_ treatment (Figure 1K,L). These findings demonstrate that H_2_O_2_-induced oxidative stress downregulates NBCn1 expression at both the mRNA and protein levels, thereby diminishing membrane localization of NBCn1 and cellular migration in the presence of NF-κB inhibition in A549 cells.

### 3.2. NF-κB Signaling Is Required for NBCn1 Activation

**Figure 2:**
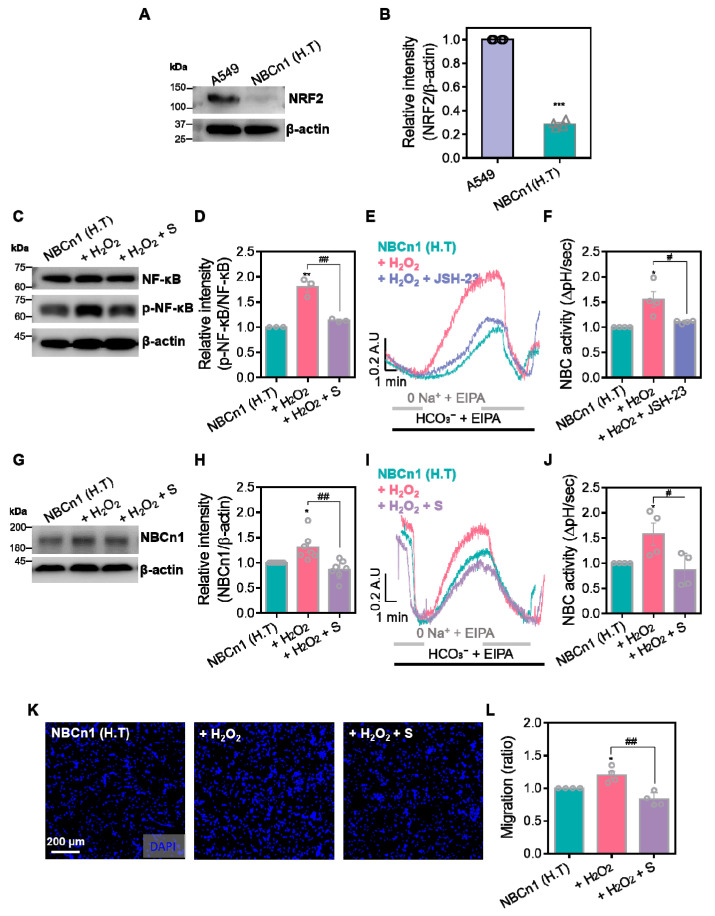
NF-κB signaling is required for NBCn1 activation. (**A**) Protein expression levels of NRF2 in A549 and NBCn1overexpressing HEK293T [NBCn1 (H.T)] cells. β-actin was used as a loading control. (**B**) Relative intensity of NRF2 normalized to β-actin. Data are presented as mean ± SEM (*n* = 4, *** *p* < 0.001 vs. A549). (**C**) Protein expression levels of total NF-κB and p-NF-κB in NBCn1 (H.T) cells treated with H_2_O_2_ (10 μM, 1 h) with or without S0859 (S; 40 μM, 1 h). β-actin was used as a loading control. (**D**) Relative intensity of p-NF-κB normalized to total NF-κB. Data are presented as mean ± SEM (*n* = 3, ** *p* < 0.01 vs. control; ## *p* < 0.01 vs. H_2_O_2_). (**E**) BCECF-based intracellular pH traces of NBC activity in NBCn1 (H.T) cells treated with H_2_O_2_ with or without JSH-23 (20 μM, 1 h). (**F**) NBC activity was assessed by measuring the recovery rate of pH_i_ (ΔpH/sec). Data are presented as mean ± SEM (*n* = 4, * *p* < 0.05 vs. control; # *p* < 0.05 vs. H_2_O_2_). (**G**) Protein expression levels of NBCn1 in NBCn1 (H.T) cells treated with H_2_O_2_ with or without S0859. β-actin was used as a loading control. (**H**) Relative intensity of NBCn1 normalized to that of β-actin. Data are presented as mean ± SEM (*n* = 7, * *p* < 0.05 vs. control; ## *p* < 0.01 vs. H_2_O_2_). (**I**) BCECF-based intracellular pH traces of NBC activity in NBCn1 (H.T) cells treated with H_2_O_2_ in the presence or absence of S0859. (**J**) NBC activity was assessed by measuring the recovery rate of pH_i_ (ΔpH/sec). Data are presented as mean ± SEM (*n* = 4, * *p* < 0.05 vs. control; # *p* < 0.05 vs. H_2_O_2_). (**K**) Representative images of transwell migration assays in NBCn1 (H.T) cells treated with H_2_O_2_ with or without S0859. Cells were stained with DAPI. Scale bar = 200 μm. (**L**) Analysis of cellular migration is shown as a relative migration ratio. Data are presented as mean ± SEM (*n* = 4, * *p* < 0.05 vs. control; ## *p* < 0.01 vs. H_2_O_2_).

To verify the relation between the NF-κB signaling and NBCn1 without the effect of NRF2, NBCn1-overexpressed HEK293T [NBCn1 (H.T)] cells were subjected to oxidative stress by H_2_O_2_ with or without NBC inhibitor S0859. Noncancerous H.T. cells expressed a low level of NRF2 (Figure 2A,B). Western blotting analysis showed that H_2_O_2_ treatment enhanced the p-NF-κB, whereas its phosphorylation was suppressed by S0859 treatment in NBCn1 (H.T) cells (Figure 2C,D). To determine whether NF-κB signaling contributes to NBCn1 function, NBCn1 (H.T) cells were treated with the NF-κB inhibitor JSH-23 [17] prior to H_2_O_2_ exposure. H_2_O_2_–evoked NBC transporter activity was reduced by JSH-23 treatment in NBCn1 (H.T) cells (Figure 2E,F). H_2_O_2_–mediated NF-κB phosphorylation in NBCn1 (H.T) cells did not align with the findings in A549 cells. To verify these results, we performed the Western blot analysis, NBC activity, and migration assay. The NBCn1 protein levels were elevated by H_2_O_2_ treatment, whereas its expression was reduced upon co-treatment with S0859 in NBCn1 (H.T) cells (Figure 2G,H). Similarly, NBC transporter activity was enhanced by H_2_O_2_ treatment, whereas its activity was attenuated by co-treatment of S0859 in NBCn1 (H.T) cells (Figure 2I,J). Expression of NBCn1 was functionally verified by transwell migration assays. Migration assays showed that H_2_O_2_ increased the migratory capacity of NBCn1 (H.T) cells, while S0859 treatment reversed this effect (Figure 2K,L). These results demonstrate that NF-κB signaling is required for the NBC activation.

### 3.3. NRF2 Is Required for the Maintenance of NBCn1 Activity in A549 Cells

**Figure 3:**
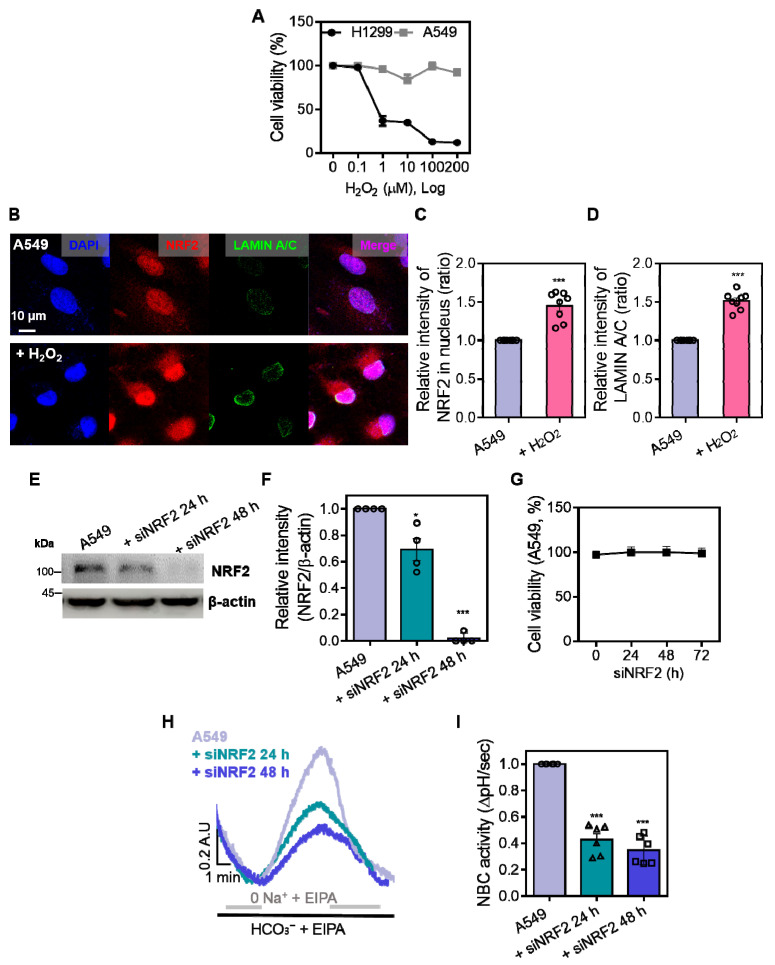
NRF2 is required for the maintenance of NBCn1 activity in A549 cells. (**A**) Cell viability of A549 and H1299 cells after treatment with increasing concentrations of H_2_O_2_ (0–200 μM, 24 h), assessed by MTT assay. (**B**) Immunofluorescence staining of NRF2 (red), LAMIN A/C (green), and nuclei (DAPI, blue) in A549 cells treated with or without H_2_O_2_ (10 μM, 1 h). Scale bar = 10 μm. (**C**) Relative intensity of NRF2 fluorescence in the nucleus. Data are presented as mean ± SEM (*n* = 8, *** *p* < 0.001 vs. control). (**D**) Relative intensity of LAMIN A/C fluorescence in the indicated condition. Data are presented as mean ± SEM (*n* = 8, *** *p* < 0.001 vs. control). (**E**) Protein expression levels of NRF2 in A549 cells transfected with siNRF2 for 24 h or 48 h. β-actin was used as a loading control. (**F**) Relative intensity of NRF2 protein normalized to β-actin. Data are presented as mean ± SEM (*n* = 4, * *p* < 0.05, *** *p* < 0.001 vs. control). (**G**) Cell viability following siNRF2 transfection for the indicated time periods (0–72 h). (**H**) BCECF-based intracellular pH traces of NBC activity in A549 cells transfected with siNRF2 for 24 h or 48 h, assessed by monitoring intracellular pH recovery following acidification. (**I**) NBC activity was evaluated by measuring the recovery rate of pH_i_ (ΔpH/sec). Data are presented as mean ± SEM (*n* = 6, *** *p* < 0.001 vs. control).

NRF2 is considered an endogenous component of the oxidative stress defense system [18]. We determined the cell viability following H_2_O_2_ exposure in A549 cells and low NRF2-expressed H1299 cells [19]. H1299 cells were more vulnerable to H_2_O_2_-induced cytotoxicity than A549 cells, indicating that A549 cells possessed a defensive system against oxidative stress (Figure 3A). To confirm whether oxidative stress induces NRF2 activation, immunofluorescence staining was performed to assess the nuclear translocation of NRF2. H_2_O_2_ treatment increased nuclear accumulation of NRF2 in A549 cells (Figure 3B,C), addressing nuclear translocation of NRF2 in response to oxidative stress. In addition, expression of LAMIN A/C, a nuclear envelope marker associated with cellular structure and migration [20], was enhanced in the presence of H_2_O_2_ (Figure 3B,D), suggesting that enhanced LAMIN A/C expression reflected reduced cellular migration. To investigate the relationship of NRF2 with NBCn1, the functional contribution of NRF2 was determined in NRF2 depletion using siRNA-NRF2 (siNRF2). Western blotting confirmed the efficacy of NRF2, and the experimental condition was selected at 48 h, which showed the reduced NRF2 expression (Figure 3E,F). Cell viability was not affected by siNRF2 treatment up to 72 h (Figure 3G). We next examined NBC activity in siNRF2-transfected A549 cells. NRF2 knockdown resulted in a reduction in NBC activity in A549 cells (Figure 3H,I). These findings demonstrate that NRF2 is involved in the maintenance of NBCn1 function in A549 cells.

### 3.4. Enhanced Phosphorylated NF-κB Expression Is Required for NBC Activation in A549 Cells

**Figure 4:**
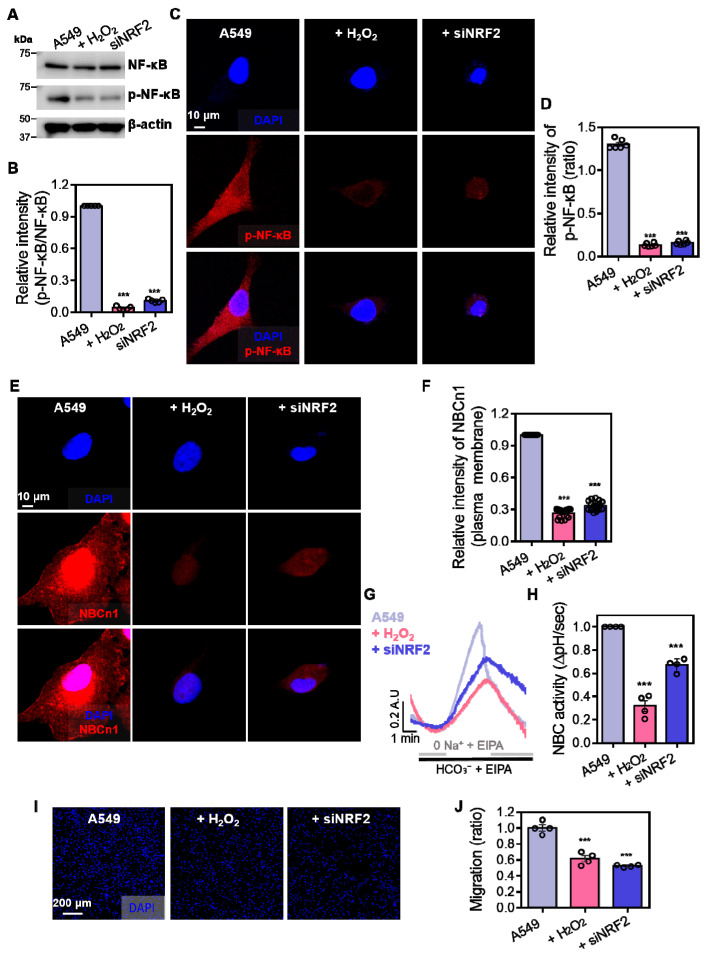
Enhanced phosphorylated NF-κB expression is required for NBC activation in A549 cells. (**A**) Protein expression levels of total NF-κB and p-NF-κB in A549 cells treated with H_2_O_2_ (10 μM, 1 h) or transfected with siNRF2 for 48 h. β-actin was used as a loading control. (**B**) Relative intensity of p-NF-κB normalized to total NF-κB. Data are presented as mean ± SEM (*n* = 6, *** *p* < 0.001 vs. control). (**C**) Immunofluorescence staining of p-NF-κB (red) and nuclei (DAPI, blue) in A549 cells treated with H_2_O_2_ or transfected with siNRF2 for 48 h. Scale bar = 10 μm. (**D**) Relative fluorescence intensity of nuclear p-NF-κB. Data are presented as mean ± SEM (*n* = 6, *** *p* < 0.001 vs. control). (**E**) Immunofluorescence staining of NBCn1 (red) and nuclei (DAPI, blue) in A549 cells treated with H_2_O_2_ or transfected with siNRF2 for 48 h. Merged images show colocalization (pink) of NBCn1 and nuclei. Scale bar = 10 μm. (**F**) Relative fluorescence intensity of plasma membrane-localized NBCn1. Data are presented as mean ± SEM (*n* = 12, *** *p* < 0.001 vs. control). (**G**) BCECF-based intracellular pH traces of NBC activity in A549 cells treated with H_2_O_2_ or transfected with siNRF2 for 48 h, measured by pH_i_ recovery following acidification. (**H**) NBC activity was assessed by measuring the recovery rate of pH_i_ (ΔpH/sec). Data are presented as mean ± SEM (*n* = 4, *** *p* < 0.001 vs. control). (**I**) Representative images of transwell migration assays in A549 cells treated with H_2_O_2_ or transfected with siNRF2 for 48 h. Cells were stained with DAPI. Scale bar = 200 μm. (**J**) Analysis of cellular migration is shown as a relative migration ratio. Data are presented as mean ± SEM (*n* = 4, *** *p* < 0.001 vs. control).

Our results showed that NF-κB signaling and NRF2 were involved in the maintenance of NBCn1 function. To verify the relationship between p-NF-κB and NRF2, Western blot analysis of p-NF-κB was performed in NRF2-depleted A549 cells. Treatment of siNRF2 reduced the p-NF-κB expression as shown in H_2_O_2_-mediated p-NF-κB suppression (Figure 4A,B). These results were consistent with immunofluorescence imaging data, which showed the reduced expression of p-NF-κB in H_2_O_2_- or siNRF2-treated cells (Figure 4C,D). Immunofluorescence analysis demonstrated reduced NBCn1 expression following H_2_O_2_ or siNRF2 treatment (Figure 4E,F). Consistent with expression data of NBCn1, H_2_O_2,_ and siNRF2 treatments reduced NBCn1 activity (Figure 4G,H) and reduced cellular migration in A549 cells (Figure 4I,J). These results indicate that NRF2 depletion attenuates NF-κB signaling, NBCn1 expression, and subsequent migratory ability in A549 cells.

### 3.5. NBC Inhibitor Attenuates NBC Activity and Migratory Property Through the NF-κB Dysregulation in the Presence of H_2_O_2_

**Figure 5:**
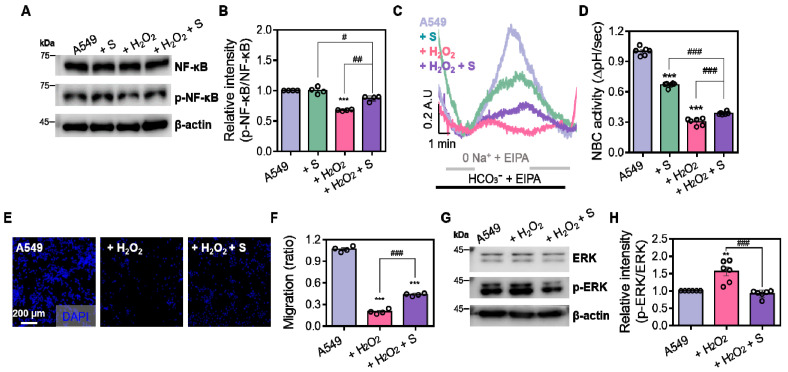
NBC inhibitor attenuates NBC activity and migratory property through the NF-κB dysregulation in the presence of H_2_O_2_. (**A**) Protein expression levels of total NF-κB and p-NF-κB in A549 cells treated with S0859 (S; 40 μM), H_2_O_2_ (10 μM), or both for 1 h. β-actin was used as a loading control. (**B**) Relative intensity of p-NF-κB/total NF-κB. Data are presented as mean ± SEM (*n* = 4, *** *p* < 0.001 vs. control; # *p* < 0.05, ## *p* < 0.01 vs. H_2_O_2_). (**C**) BCECF-based intracellular pH traces of pH_i_ recovery to assess NBC activity in A549 cells treated with H_2_O_2_ in the presence/absence of S0859. (**D**) NBC activity was calculated by measuring the recovery rate of pH_i_ (ΔpH/sec). Data are presented as mean ± SEM (*n* = 7–15, *** *p* < 0.001 vs. control; ### *p* < 0.001 vs. H_2_O_2_). (**E**) Representative images of transwell migration assays in A549 cells treated with H_2_O_2_ (10 μM) or H_2_O_2_ (10 μM) + S0859 (40 μM) for 1 h. Cells were stained with DAPI. Scale bar = 200 μm. (**F**) Relative migration ratio measured by the transwell assay in A549 cells treated as indicated. Data are presented as mean ± SEM (*n* = 4, *** *p* < 0.001 vs. control; ### *p* < 0.001 vs. H_2_O_2_). (**G**) Protein expression levels of total ERK and p-ERK in A549 cells treated with H_2_O_2_ in the presence/absence of S0859. β-actin was used as a loading control. (**H**) Relative intensity of p-ERK/total ERK. Data are presented as mean ± SEM (*n* = 6, ** *p* < 0.01 vs. control; ### *p* < 0.001 vs. H_2_O_2_).

To verify the modulatory effect of S0859 in the presence of oxidative stress on NF-κB signaling in A549 cells, the NBC inhibitor S0859 was treated in the presence or absence of oxidative stress. Expression of p-NF-κB was reduced by S0859 treatment in the presence of H_2_O_2_ treatment (Figure 5A,B). Similarly, S0859 inhibited NBC activity and migration in the presence or absence of H_2_O_2_ (Figure 5C–F). The effect of S0859 on NBC activity in the presence of H_2_O_2_ was observed in additive inhibition compared to that of S0859 alone (Figure 5D). In addition, to evaluate the modulatory effect of S0859 on ERK signaling, whether the ERK signaling is involved in the regulation of NBC activity in A549 cells, S0859 was treated in the presence or absence of oxidative stress. Oxidative stress-induced p-ERK expression was reduced in the presence of S0859 (Figure 5G,H), indicating that S0859 induces the dysregulated ERK signaling. These results indicate that NBC inhibitor S0859 induces ERK dysregulation and oxidative stress-mediated NBC dysregulation through the inhibited NF-κB signaling.

### 3.6. S0859 Dysregulates NRF2 Expression and Oxidative Stress Defense System in A549 Cells

**Figure 6:**
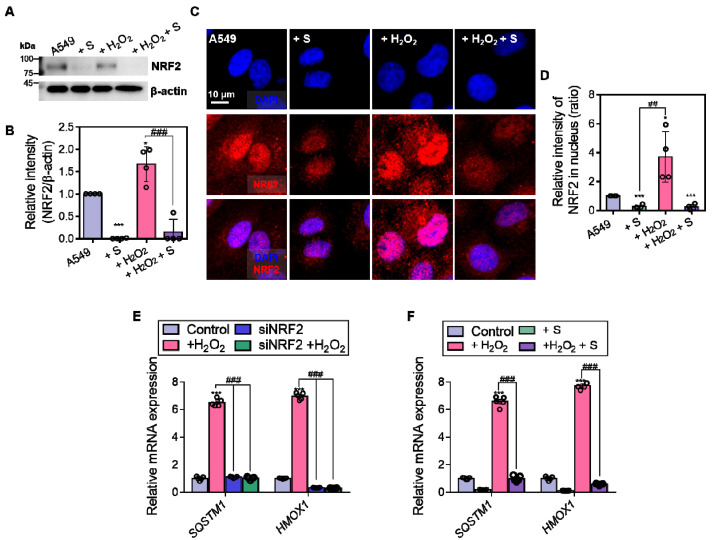
S0859 dysregulates NRF2 expression and the oxidative stress defense system in A549 cells. (**A**) Protein expression levels of NRF2 in A549 cells treated with S0859 (S; 40 μM), H_2_O_2_ (10 μM), or both for 1 h. β-actin was used as a loading control. (**B**) Relative intensity of NRF2/β-actin. Data are presented as mean ± SEM (*n* = 4, * *p* < 0.05, *** *p* < 0.001 vs. control; ### *p* < 0.001 vs. H_2_O_2_). (**C**) Immunofluorescence staining of NRF2 (red) and nuclei (DAPI, blue) in A549 cells treated with S0859 (40 μM), H_2_O_2_ (10 μM), or both for 1 h. Merged images show colocalization of NRF2 and nuclei, where pink or purple indicates overlapping fluorescence signals. Scale bar = 10 μm. (**D**) Relative fluorescence intensity of nuclear NRF2. Data are presented as mean ± SEM (*n* = 4, * *p* < 0.05, *** *p* < 0.001 vs. control; ## *p* < 0.01 vs. H_2_O_2_). (**E**) mRNA expression levels of *SQSTM1* and *HMOX1* in A549 cells treated with H_2_O_2_ (10 μM, 1 h), with or without siNRF2 transfection. Data are presented as mean ± SEM (*n* = 5, *** *p* < 0.001 vs. control; ### *p* < 0.001 for indicated comparisons). (**F**) mRNA expression levels of *SQSTM1* and *HMOX1* in A549 cells treated with S0859, H_2_O_2_, or both. Data are presented as mean ± SEM (*n* = 5, *** *p* < 0.001 vs. control; ### *p* < 0.001 for indicated comparisons).

To determine whether S0859 modulates antioxidant gene expression under oxidative stress, NRF2 expression and its nuclear localization were examined in A549 cells. S0859 treatment reduced NRF2 expression (Figure 6A,B). NRF2 was observed in cytoplasmic location in the presence of S0859, whereas H_2_O_2_ stimulation triggered nuclear translocation of NRF2 (Figure 6C,D), suggesting that H_2_O_2_ stimulation induces oxidative stress defense response, whereas S0859 dysregulates the H_2_O_2_-mediated oxidative stress defense system. Quantitative RT-PCR revealed that H_2_O_2_ increased mRNA expression of the NRF2 target genes *SQSTM1* (encodes p62) and *HMOX1* (encodes HO-1) [21,22], whereas this induction was nearly abolished by both NRF2 knockdown (Figure 6E) and S0859 treatment (Figure 6F), indicating that NRF2 is essential for proper antioxidant gene expression in response to oxidative stress, and treatment of the NBC inhibitor impairs NRF2-mediated oxidative stress defense system.

## 4. Discussion

In this study, we determined that H_2_O_2_-induced oxidative stress induced dysregulated NBCn1 expression and activity through NF-κB inhibition and subsequently attenuated cellular migration in A549 cells. H_2_O_2_ stimulation induced nuclear accumulation of NRF2 to enhance expression of oxidative stress defense genes. Phosphorylation of NF-κB was involved in the NBC activation and increased migration in A549 cells. Application of NBC inhibitor S0859 induced oxidative stress-mediated NBC dysregulation through dysregulated NF-κB activation. Treatment of S0859 impaired ERK activation and nuclear translocation of NRF2, as well as expression of oxidative stress defense genes, even in the presence of oxidative stimulation in A549 cells. Moreover, oxidative stress stimulation in the presence of NBC inhibitor S0859 did not recover NBC activity and the NRF2-mediated oxidative stress defense system in A549 cells.

NRF2 activation is mediated by ERK activation [23,24]. Our results showed that S0859 treatment inhibited phosphorylation of ERK, thereby inducing NRF2 inhibition. On the other hand, NBC isoforms consist of both electroneutral type (NBCn: electroneutral stoichiometry) and electrogenic type (NBCe: electrogenic stoichiometry) [25]. Although the activity of NBCe1 is revealed to be reactive oxygen species and an ERK-dependent pathway in cat cardiomyocytes [26], differential regulation of NBC activity in each NBC isotype or its tissues/species-specific regulation should be precisely verified. Our results supported that ERK signaling is involved in the modulation of NRF2 rather than that of NBC activity.

Crosstalk between NRF2 and NF-κB has been known, where NRF2 inhibits NF-κB signaling as a negative feedback loop [27,28,29], suggesting that S0859 revealed a double inhibitory effect on the modulation of NRF2 through both ERK inhibition and NF-κB activation. In this study, although S0859 treatment maintained the expression of p-NF-κB, which has been known to induce NRF2 inhibition, oxidative stress reduced the expression of p-NF-κB, thereby reducing NBC activity and cellular migration. Jin et al. [30] Reported that traumatic brain injury-exposed NRF2-null mice revealed higher NF-κB activation than wild-type mice. Moreover, the anti-inflammatory cyclo (His-Pro) dipeptide enhanced NRF2 signaling through the inhibition of NF-κB [31]. Thus, to address the antagonistic regulation between NRF2 and NF-κB through the NRF2 depletion, careful consideration should be given.

**Figure 7:**
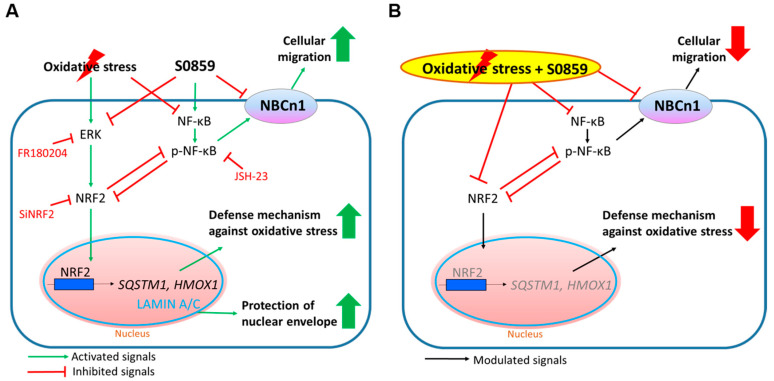
Schematic model illustrates the regulatory effects of S0859 and oxidative stress signaling in A549 cells. (**A**) Under oxidative stress (H_2_O_2_ stimulation), NRF2 is activated via ERK signaling and translocated to the nuclear fraction, thereby promoting antioxidant gene expression (e.g., *SQSTM1* and *HMOX1*) and suppressing NBCn1 through the inhibition of NF-κB activation. Moreover, oxidative stress induced the enhancement of nuclear envelope protein LAMIN A/C to protect the nuclear envelope. NBC inhibitor S0859 inhibited ERK, whereas maintaining NF-κB signaling, and these signaling events mediated NRF2 suppression, reduced antioxidant response, NBCn1 expression, and cellular migration. (**B**) In the presence of oxidative stress and S0859, NBC activity-mediated cellular migration and NRF2-mediated oxidative stress defense system were dysregulated in lung cancer cells. Collectively, treatment of S0859 inhibited the defense mechanism against oxidative stress and cellular migration, suggesting that S0859 revealed the potential effect on NRF2 inhibition as a lung cancer treatment strategy.

Based on our results, a schematic model was proposed to describe the regulatory effects of S0859 on oxidative stress-mediated signaling, including the regulation of migratory module NBCn1 and oxidative stress defense genes in A549 NSCLC cells, which are a high level of NRF2 and Keap1 mutated cell line (Figure 7). Under oxidative stress induced by H_2_O_2_, activation of ERK and dephosphorylated NF-κB promoted the nuclear translocation of NRF2, leading to the upregulation of antioxidant genes such as *SQSTM1* and *HMOX1*. These events collectively contribute to the activation of the cellular oxidative stress defense mechanism. In contrast, treatment with S0859 disrupted this protective response through the suppression of NRF2. Specifically, S0859 treatment inhibited ERK phosphorylation, thereby attenuating nuclear accumulation of NRF2. As an NBC inhibitor, S0859 reduced NBCn1 activity and cellular migration. The schematic model highlighted a critical role for NBCn1 in coordinating redox-responsive signaling and suggested that S0859 disrupted NRF2-dependent cyto-protective responses by interfering with both ERK and NF-κB signaling pathways. Thus, modulation of NRF2-mediated redox resistance against antioxidant drugs would be considered by the S0859 co-treatment as an application strategy to diminish redox resistance in cancer cells.

Consistent with this observation, several recent studies have also explored NRF2 inhibition as a means to overcome redox-associated drug resistance in lung cancer cells. For instance, recent work with a combined approach of metformin and cisplatin inhibits Nrf2 through the inhibition of ERK1/2 and enhances proteasomal degradation in lung cancer [32]. Moreover, liposome-launched quinacrine enhances cisplatin-mediated cell death through the inhibition of NRF2 expression in A549 lung cancer cells [33]. Similarly, combinations of naturally driven phytochemicals have been suggested to improve the modulating effects on NRF2 and NF-κB signaling to prevent cancer survival [34].

In this study, we addressed that S0859 could be a promising therapeutic anti-cancer drug as a potential NRF2 inhibitor in oxidative stress-induced NSCLC therapy. Moreover, we provided the experimental potential of S0859 as a supportive drug to apply the combinational therapy against cancers. Unfortunately, a limitation of the present study is that cytotoxicity was evaluated exclusively in malignant cell lines, which precludes definitive conclusions regarding the therapeutic selectivity of S0859. Future studies will therefore include comprehensive assessments of cytotoxicity and cardiotoxicity [35], as well as the influence of metabolic syndromes and altered redox profiles in relevant non-malignant human cell types, to better define the therapeutic window of S0859.

## Data Availability

The data supporting the findings of this study, including confocal microscopy images and quantitative analysis results, are available from the corresponding author upon reasonable request.
